# 

**Published:** 1908-10-24

**Authors:** 


### BOOKS RECEIVED.

J. B. Lippincott Co.

"International Clinics." 18th Series. Vol. III.

Kegan, Paul, Trench, Trubner and Co., Ltd.

"Tutonish." By Elias Molee, Ph.D.

John Bale, Sons and Danielsson, Ltd.

"When to Operate in Inflammation of the Appendix." By C. Mansell Moullin, M.D.

J. and A. Churchill.

"First Lines in Dispensing." By E. W. Lucas, F.I.C., F.C.S.

Archibald Constable and Co., Ltd.

"From an Easy Chair." By Sir E. Ray Lankester, K.C.B., F.R.S.

Wm. Green and Sons, Edinburgh.

"Diet in Infancy." By A. Dingwall-Fordyce, M.D., F.R.C.P. Ed.

John Heywood, Ltd., Manchester.

"Wastage of Child Life." By J. Johnston, M.D.

Longmans, Green and Co.

"How to Nurse Sick Children." By Chas. West, M.D.

S. W. Partridge and Co.

"The Zenana, or Woman's Work in India." Vol. 15. 1908.

J. Thin, Edinburgh.

"Pharmacopœia of the Royal Infirmary, Edinburgh."

The University Tutorial Press.

"The London University Guide and University Correspondence Calendar, 1909."

"Matriculation Directory." No. 50. September 1908.

### THE GRADUATES' MEDICAL SCHOOLS.

DIARY FOR WEEK OCT. 26 TO OCT. 31.

**NATIONAL HOSPITAL FOR THE PARALYSED AND EPILEPTIC, Queen Sq., Bloomsbury, W.C.**

**Oct. 27,** Dr. Tooth, **Muscular Atrophy.**

**Oct. 30,** Sir V. Horsley, **Surgery of the Nervous System.**

**THE POST-GRADUATE COLLEGE, West London Hospital, Hammersmith, W.**

**At 12 noon.**

**Oct. 26,** Dr. Low, **Pathological Demonstration.**

**At 12.15 p.m.**

**Oct. 28 and 30,** Dr. Pritchard, **Practical Medicine.**

**At 5 p.m.**

**Oct. 26,** Dr. Davis, **Common Diseases of the Pharynx.**

**Oct. 27,** Dr. Low, **Insects as Carriers of Disease, especially in Reference to the Tropics.**

**Oct. 28,** Dr. Beddard, **Medicine, II.**

**Oct. 29,** Mr. Edwards, **Rectal Cases.**

**Oct. 30,** Mr. Pardoe, **The Treatment of Urethral Stricture.**

**THE THROAT HOSPITAL, Golden Square, W.**

**At 5.30 p.m.**

**Oct. 26,** Mr. Parker, **Methods of Local Treatment.**

**Oct. 29,** Mr. Rose, **Acute Rhinitis.**

**NORTH-EAST LONDON POST-GRADUATE COLLEGE, Prince of Wales's General Hospital, Tottenham, N.**

**At 4.30 p.m.**

**Oct. 27,** Dr. G. N. Meachen, **Demonstration of Skin Cases.**

**Oct. 29,** Mr. H. W. Carson, **Modern Methods of Treatment of Obstruction of the Large Intestine.**

**ST. JOHN'S HOSPITAL FOR DISEASES OF THE SKIN, Leicester Square, W.C.**

**At 6 p.m.**

**Oct. 29,** Chesterfield Lecture, **The Treatment of Eczema in all its Forms.**

**MOUNT VERNON HOSPITAL, Fitzroy Square, W.**

**At 5 p.m.**

**Oct. 28,** Sir James Barr, **Mitral Stenosis.**

**LONDON SCHOOL OF CLINICAL MEDICINE, Seamen's Hospital, Greenwich, S.E.**

**At 3.30 p.m.**

**Oct. 28,** Mr. Cargill, **The Examination of the Field of Vision and its Importance.**

**At 2.30 p.m.**

**Oct. 29,** Dr. Rankin, **Acromegaly** (with illustrative cases).

**MEDICAL GRADUATES' COLLEGE AND POLYCLINIC, 22 Chenies Street, W.C.**

**Oct. 26,** Dr. Purves Stewart, **The Prognosis and Treatment of Infantile Paralysis.**

**Oct. 27,** Dr. J. Mackenzie, **The X Disease.**

**Oct. 28,** Dr. A. J. Whiting, **Two Important Angio-Neuroses.**

**Oct. 29,** Dr. F. M. Sandwith, **Malaria—Its Prevention and Treatment.**

### THE ROYAL COLLEGE OF PHYSICIANS OF LONDON.

THE Bradshaw Lecture will be given before the Royal College of Physicians of London by Dr. W. Pasteur on November 3, at 5 P.M., the subject being Massive Collapse of the Lung. The Fitz-Patrick Lectures, which will deal this year with the History of Neurology, will be given on November 5 and 10, at the same hour, by Dr. Leonard Guthrie. The Horace Dobell Lecture by Dr. Leonard S. Dudgeon, F.R.C.P., will be given on November 12, the subject being the Latent Persistence and the Reactivation of Pathogenic Bacteria in the Body. Dr. F. W. Pavy, F.R.S., will give a course of three lectures on the Pathology and Treatment of Diabetes Mellitus, Viewed by the Light of Present-day Knowledge, at the College on November 17, 19, and 24, at 5 P.M. on each day.

### ANSWERS TO CORRESPONDENTS.

H. HAYNES HUTCHINSON.—The principal hospital in the city of Vancouver, British Columbia, is the Vancouver General Hospital, with 150 beds. St. Paul's Hospital is also in the city. We have no particulars of any nursing homes there. There is a nurses' training school, with home attached, in connection with the Vancouver General Hospital.

### Subscriptions.

The rate of annual subscriptions to THE HOSPITAL, either direct from the Office or through agents, is :—

| | |
|---|---|
| For the United Kingdom ... ... ... | 15s. a year |
| To the Colonies and Abroad ... ... | 17s. a year |

inclusive of postage in each case.

